# Supplementary material for: Sleeping pattern and activities of daily living modulate protein expression in AMD
Source: PLoS One. 2021 Jun 1;16(6):e0248523. doi: 10.1371/journal.pone.0248523 (PMC8168906; doi:10.1371/journal.pone.0248523)
Supplement: S1 File — (DOCX) [file pone.0248523.s001.docx]

| genotypes | | Group code | | | | Unadjusted p-value | | | Multivariate adjusted for age, sex, food habit, smoking, alcohol | | | | AIC | BIC |
| --- | --- | --- | --- | --- | --- | --- | --- | --- | --- | --- | --- | --- | --- | --- |
|  |  | **AMD** | | **Controls** | | **p-value** | **OR** | **95% CI** | **p-value** | **OR** | **95% CI** | |  |  |
| S1 File  Sleeping pattern and activities of daily living modulate protein expression in AMD (PONE-D-20-29337R1)  rs6795735  (ADAMTS9) | TT | 114 | 52.5% | 57 | 61.3% | Ref |  |  |  |  |  |  |  |  |
|  | CC | 20 | 9.2% | 3 | 3.2% | **0.060** | 0.300 | 0.086-1.052 | **0.013** | 0.049 | 0.004-0.535 | | 231.4 | 264.1 |
|  | CT | 83 | 38.2% | 33 | 35.5% | 0.382 | 0.795 | 0.476-1.329 | 0.785 | 0.911 | 0.465-1.783 | |  |  |
|  | T | 311 | 71.7% | 167 | 81.1% | Ref |  |  |  |  |  | |  |  |
|  | C | 123 | 28.3% | 39 | 18.9% | **.011** | .011 | .393-.883 |  |  |  | |  |  |
|  |  | **AREDS 3 & 4 (dry)** | | **AREDS 5 (wet)** | |  |  |  |  |  |  |  |  |  |
| rs6795735  (ADAMTS9) | TT | 36 | 50.0% | 78 | 53.8% | Ref | | |  | | | |  |  |
|  | CC | 9 | 12.5% | 11 | 7.6% | 0.245 | 0.564 | 0.215-1.481 | 0.185 | 0.497 | 0.176-1.398 | |  |  |
|  | CT | 27 | 37.5% | 56 | 38.6% | 0.888 | 0.957 | 0.522-1.754 | 0.851 | 0.941 | 0.499-1.774 | |  |  |
|  | T | 99 | 68.8% | 212 | 73.1% | Ref |  |  |  |  |  | |  |  |
|  | C | 45 | 31.3% | 78 | 26.9% | .343 | **.**809 | .523-1.254 |  |  |  | |  |  |
|  |  | **AMD** | | **Controls** | |  |  |  |  |  |  | |  |  |
| rs5749482  (TIMP-3) | GG | 184 | 81.1% | 77 | 67.5% | Ref |  |  |  |  |  | |  |  |
|  | CC | 5 | 2.2% | 6 | 5.3% | .090 | 2.868 | .850-9.677 | .397 | 2.178 | .360-13.195 | | 288.8 | 322.4 |
|  | GC | 38 | 16.7% | 31 | 27.2% | **.016** | 1.949 | 1.132-3.358 | .146 | 1.657 | .839-3.274 | |  |  |
|  | G | 406 | 89.4% | 185 | 81.1% | Ref |  |  |  |  |  | |  |  |
|  | C | 48 | 10.6% | 43 | 18.9% | **0.003** | 1.966 | 1.258-3.073 |  |  |  | |  |  |
|  |  | **AREDS 3 & 4 (dry)** | | **AREDS 5 (wet)** | |  |  |  |  |  |  | |  |  |
| rs5749482  (TIMP-3) | GG | 53 | 72.6% | 131 | 85.1% | ref |  |  |  |  |  | |  |  |
|  | CC | 4 | 5.5% | 1 | 0.6% | **.043** | .101 | .011-.926 | **.032** | .082 | .008-.803 | |  |  |
|  | GC | 16 | 21.9% | 22 | 14.3% | .110 | .556 | .271-1.141 | .176 | .598 | .284-1.259 | |  |  |
|  | G | 122 | 83.6% | 284 | 92.2% | Ref |  |  |  |  |  | |  |  |
|  | C | 24 | 16.4% | 24 | 7.8% | **.005** | .430 | .235-.786 |  |  |  | |  |  |
|  |  | **AMD** | | **Controls** | |  |  |  |  |  |  | |  |  |
| rs11200638  (HTRA1) | GG | 42 | 20.7% | 42 | 36.5% | Ref |  |  |  |  |  | |  |  |
|  | AA | 100 | 49.3% | 27 | 23.5% | **<.0001** | .270 | .148-.493 | **.002** | .327 | .161-.668 | | 291.1 | 324 |
|  | GA | 61 | 30.0% | 46 | 40.0% | .335 | .754 | .425-1.339 | .945 | .976 | .490-1.944 | |  |  |
|  | G | 145 | 35.7% | 130 | 56.5% | Ref |  |  |  |  |  | |  |  |
|  | A | 261 | 64.3% | 100 | 43.5% | **<0.0001** | .427 | .307-.595 |  |  |  | |  |  |
|  |  | **AREDS 3 & 4 (dry)** | | **AREDS 5 (wet)** | |  |  |  |  |  |  | |  |  |
| rs11200638  (HTRA1) | GG | 20 | 29.9% | 22 | 16.2% | Ref |  |  |  |  |  | |  |  |
|  | AA | 24 | 35.8% | 76 | 55.9% | **.006** | 2.879 | 1.347-6.154 | **.006** | 3.074 | 1.376-6.867 | |  |  |
|  | GA | 23 | 34.3% | 38 | 27.9% | .317 | 1.502 | .677-3.332 | .583 | 1.272 | .539-3.00 | |  |  |
|  | G | 73 | 50.7% | 82 | 30.1% | Ref |  |  |  |  |  | |  |  |
|  | A | 71 | 49.3% | 190 | 69.9% | **<0.0001** | 2.382 | 1.570-3.615 |  |  |  | |  |  |
|  |  | **AMD** | | **Controls** | |  |  |  |  |  |  | |  |  |
| rs769449  (APOE) | GG | 200 | 88.9% | 94 | 77.7% | Ref |  |  |  |  |  | |  |  |
|  | AA | 0 | 0.0% | 0 | 0.0% |  |  |  |  |  |  | |  |  |
|  | AG | 25 | 11.1% | 27 | 22.3% | **0.006** | 2.298 | 1.265-4.173 | **0.003** | 3.355 | 1.509-7.462 | | 286 | 315.9 |
|  | G | 425 | 94.4% | 215 | 88.8% | Ref |  |  |  |  |  | |  |  |
|  | A | 25 | 5.6% | 27 | 11.2% | **0.008** | 2.135 | 1.210-3.760 |  |  |  | |  |  |
|  |  | **AREDS 3 & 4 (dry)** | | **AREDS 5 (wet)** | |  |  |  |  |  |  | |  |  |
| rs769449  (APOE) | GG | 63 | 86.3% | 137 | 90.1% | Ref |  |  |  |  |  | |  |  |
|  | AA | 0 | 0.0% | 0 | 0.0% |  |  |  |  |  |  | |  |  |
|  | AG | 10 | 13.7% | 15 | 9.9% | .394 | .690 | .294-1.620 | .421 | .697 | .290-1.677 | |  |  |
|  | G | 99 | 68.8% | 289 | 95.1% | Ref |  |  |  |  |  | |  |  |
|  | A | 45 | 31.3% | 15 | 4.9% | **<0.0001** | .114 | .061-.214 |  |  |  | |  |  |
|  |  | **AMD** | | **Controls** | |  |  |  |  |  |  | |  |  |
| rs920915  (LIPC) | G | 287 | 67.7% | 101 | 57.4% | Ref |  |  |  |  |  | |  |  |
|  | C | 137 | 32.3% | 75 | 42.6% | **.016** | 1.556 | 1.084-2.233 |  |  |  | |  |  |
|  |  | **AREDS 3 & 4 (dry)** | | **AREDS 5 (wet)** | |  |  |  |  |  |  | |  |  |
|  | G | 99 | 68.8% | 289 | 95.1% | Ref |  |  |  |  |  | |  |  |
|  | A | 45 | 31.3% | 15 | 4.9% | **<0.0001** | .114 | .061-.214 |  |  |  | |  |  |

**Table 1:** Logistic regression and allele frequency distribution of genetic variants in Indian AMD. Logistic regression analysis to associate the genotype frequencies of various genetic variants with North-West Indian AMD using univariate and multivariate method. Association of allele frequencies of various genetic variants with North-West Indian AMD by univariate logistic regression.

Published: *Sharma K et al., Genomics. 2021 Jan;113(1 Pt 2):514-522. doi: 10.1016/j.ygeno.2020.09.044. Epub 2020 Sep 24.*

| Genotype | Number (frequency) | | | | Unadjusted p value | | | Multivariate analysis adjusted for age, sex | | |
| --- | --- | --- | --- | --- | --- | --- | --- | --- | --- | --- |
|  |  |  |  |  | **P-value** | **OR** | **95% CI** | **P-value** | **OR** | **95% CI** |
| ADAMTS9 rs6795735 | | | | | | | | | | |
|  | **AMD disturbed sleep** | | **AMD normal sleep** | |  |  |  |  |  |  |
| TT | 47 | 56.0% | 62 | 49.2% | Ref |  |  |  |  |  |
| CC | 4 | 4.8% | 15 | 11.9% | **.079** | .352 | .110-1.129 | **.059** | .318 | .097-1.043 |
| CT | 33 | 39.3% | 49 | 38.9% | .690 | .888 | .497-1.590 | .676 | .882 | .490-1.589 |
| TIMP3 rs5749482 | | | | | | | | | | |
|  | **AMD disturbed sleep** | | **AMD normal sleep** | |  |  |  |  |  |  |
| GG | 77 | 90.6% | 102 | 75.6% | Ref |  |  |  |  |  |
| CC | 0 | 0.0% | 5 | 3.7% | .999 | .000 | 0.000 | .999 | .000 | 0.000 |
| GC | 8 | 9.4% | 28 | 20.7% | **.023** | .378 | .163-.876 | **.012** | .334 | .141-.789 |
| HTRA1 rs11200638 | | | | | | | | | | |
|  | **AMD Alcohol** | | **AMD Never alcoholic** | |  |  |  |  |  |  |
| GG | 10 | 15.4% | 32 | 23.2% | Ref |  |  |  |  |  |
| AA | 31 | 47.7% | 69 | 50.0% | .390 | 1.438 | .629-3.287 | .193 | 1.892 | .724-4.944 |
| GA | 24 | 36.9% | 37 | 26.8% | .102 | 2.076 | .864-4.986 | **.040** | 3.029 | 1.052-8.718 |
| IER3 rs3130783 | | | | | | | | | | |
|  | **Vegetarian AMD** | | **Nonveg AMD** | |  |  |  |  |  |  |
| AA | 94 | 75.2% | 90 | 87.4% | Ref |  |  |  |  |  |
| GG | 3 | 2.4% | 0 | 0.0% | .999 | .000 | 0.000 | .999 | .000 | 0.000 |
| AG | 28 | 22.4% | 13 | 12.6% | **.048** | .485 | .236-.995 | **.057** | .491 | .237-1.020 |
| APOE rs769449 | | | | | | | | | | |
|  | **AMD Smoker** | | **AMD non smoker** | |  |  |  |  |  |  |
| GG | 61 | 81.3% | 137 | 92.6% | Ref |  |  |  |  |  |
| AA | 0 | 0.0% | 0 | 0.0% |  |  |  |  |  |  |
| AG | 14 | 18.7% | 11 | 7.4% | **.015** | 1.691 | 1.108-2.580 | **.031** | 2.907 | 1.103-7.664 |
| LIPC rs920915 | | | | | | | | | | |
|  | **Vegetarian AMD** | | **Nonveg AMD** | |  |  |  |  |  |  |
| GG | 58 | 50.9% | 42 | 43.3% | Ref |  |  |  |  |  |
| CC | 9 | 7.9% | 16 | 16.5% | **.053** | 2.455 | .990-6.087 | **.063** | 2.407 | .954-6.070 |
| GC | 47 | 41.2% | 39 | 40.2% | .646 | 1.146 | .641-2.049 | .661 | 1.143 | .630-2.071 |
|  | **AMD Smoker** | | **AMD non smoker** | |  |  |  |  |  |  |
| GG | 37 | 54.4% | 62 | 43.7% | Ref |  |  |  |  |  |
| CC | 10 | 14.7% | 15 | 10.6% | .809 | 1.117 | .455-2.742 | .989 | .993 | .373-2.643 |
| GC | 21 | 30.9% | 65 | 45.8% | **.060** | .541 | .286-1.025 | **.050** | .500 | .250-1.001 |
|  | **AMD disturbed sleep** | | **AMD normal sleep** | |  |  |  |  |  |  |
| GG | 35 | 42.7% | 60 | 48.8% | Ref |  |  |  |  |  |
| CC | 17 | 20.7% | 8 | 6.5% | **.007** | 3.643 | 1.426-9.307 | **.004** | 4.047 | 1.551-10.558 |
| GC | 30 | 36.6% | 55 | 44.7% | .829 | .935 | .508-1.720 | .817 | .929 | .498-1.734 |

Table2 : Association of covariates with genotype frequencies in Indian AMD patients using logistic regression analysis.

Published: *Sharma K et al., Genomics. 2021 Jan;113(1 Pt 2):514-522. doi: 10.1016/j.ygeno.2020.09.044. Epub 2020 Sep 24.*

| Proteins | Group | N | Mean | F-value | t-value | P-value |
| --- | --- | --- | --- | --- | --- | --- |
| ADAMTS9 (pg/ug) | **AMD** | **206** | **10.065** | **34.122** | **3.105** | **0.00206** |
|  | **Controls** | **142** | **2.629** |  |  |  |
| APOE (pg/ug) | **AMD** | **206** | **0.026** | **18.619** | **2.526** | **0.011939** |
|  | **Controls** | **155** | **0.0036** |  |  |  |
| B3GALTL (pg/ug) | **AMD** | **190** | **6.054** | **10.534** | **3.763** | **0.000201** |
|  | **Controls** | **125** | **3.393** |  |  |  |
| HTRA1 (pg/ug) | **AMD** | **193** | **4.45** | **13.295** | **3.823** | **0.000158** |
|  | **Controls** | **130** | **2.618** |  |  |  |
| LIPC (pg/ug) | **AMD** | **193** | **3.675** | **45.747** | **4.401** | **0.00001** |
|  | **Controls** | **149** | **1.619** |  |  |  |
| TIMP3 (pg/ug) | **AMD** | **187** | **0.061** | **18.008** | **4.239** | **0.000029** |
|  | **Controls** | **146** | **0.021** |  |  |  |
| IER3 (pg/ug) | **AMD** | **186** | **5.5** | **41.988** | **5.561** | **<0.0001** |
|  | **Controls** | **145** | **1.519** |  |  |  |
| SLC16A8 (pg/ug) | **AMD** | **187** | **0.841** | **24.551** | **-2.307** | **0.021696** |
|  | **Controls** | **131** | **1.378** |  |  |  |

Table 3: Differential expression of proteins in serum of AMD and their comparisons with controls.

Published: *Sharma K et al., Genomics. 2021 Jan;113(1 Pt 2):514-522. doi: 10.1016/j.ygeno.2020.09.044. Epub 2020 Sep 24.*
