# Supplementary material for: Sleeping pattern and activities of daily living modulate protein expression in AMD
Source: PLoS One. 2021 Jun 1;16(6):e0248523. doi: 10.1371/journal.pone.0248523 (PMC8168906; doi:10.1371/journal.pone.0248523)
Supplement: Attachment — Submitted filename: Rebuttal letter.docx [file pone.0248523.s002.docx]

PONE-D-20-29337
Sleeping pattern and activities of daily living modulate protein expression in AMD

Dear Editor

We have addressed all the comments raised by the reviewers and modified the manuscript as per journal requirements. Please see revised manuscript for your kind consideration.

Please evaluate the role of epigenetic changes in the onset and progression of AMD.
**Response**: We have added the requisite literature to support our results of the manuscript. Thanks for valuable comment.

Sincerely

Akshay Anand, PhD

Professor

Neuroscience Research lab

Department of Neurology

PGIMER, Chandigarh, India.

**Journal Requirements:**

When submitting your revision, we need you to address these additional requirements.

1. Please ensure that your manuscript meets PLOS ONE's style requirements, including those for file naming. The PLOS ONE style templates can be found at

<https://journals.plos.org/plosone/s/file?id=wjVg/PLOSOne_formatting_sample_main_body.pdf> and

<https://journals.plos.org/plosone/s/file?id=ba62/PLOSOne_formatting_sample_title_authors_affiliations.pdf>

**Response**: Thanks for comment. Manuscript has been changed as per the template provided.

2. In your Methods section, please provide additional information about the participant recruitment method and the demographic details of your participants. Please ensure you have provided sufficient details to replicate the analyses such as: a) the recruitment date range (month and year), b) a description of any inclusion/exclusion criteria that were applied to participant recruitment, c) a table of relevant demographic details, d) a statement as to whether your sample can be considered representative of a larger population, e) a description of how participants were recruited, and f) descriptions of where participants were recruited and where the research took place.

**Response** 2: a) the recruitment date range (month and year)

**Response**: The methodology has been updated as per the reviewer comment.

b). a description of any inclusion/exclusion criteria that were applied to participant recruitment.

**Response**: We have expanded the inclusion criteria of the manuscript, based on which we have recruited the participants.

c) a table of relevant demographic details

**Response**: Thanks for the comment. We have mentioned the socio-demographic characteristics of the recruited participants in Table 1, 2 and 3. Please see for your kind consideration.

d) a statement as to whether your sample can be considered representative of a larger population.

**Response**: We have added the statement as reviewer suggested. Please see ‘Recruitment of participants’ section of the manuscript.

e) a description of how participants were recruited.

**Response**: The recruitment of the participants was done as per the inclusion and exclusion criteria of the study as mentioned in the ‘inclusion criteria and recruitment of participants’ sections of the manuscript.

f) descriptions of where participants were recruited and where the research took place.

**Response**: The recruitment of participants and clinical examination were done from Department of Ophthalmology, PGIMER, Chandigarh, India and experiments were conducted in Neuroscience Research Lab, Dept of Neurology, Post Graduate Institute of medical Education and Research, Chandigarh, India. We have updated the recruitment of participants section as per the reviewer comment.

3. We suggest you thoroughly copyedit your manuscript for language usage, spelling, and grammar. If you do not know anyone who can help you do this, you may wish to consider employing a professional scientific editing service.

**Response**: We have substantially edited the manuscript with native English to enhance the readability of the same. We have also acknowledges the same in the ‘acknowledgment section’ of the manuscript. Thanks for your comment.  

Upon resubmission, please provide the following:

- The name of the colleague or the details of the professional service that edited your manuscript
- A copy of your manuscript showing your changes by either highlighting them or using track changes (uploaded as a *supporting information* file)
- A clean copy of the edited manuscript (uploaded as the new *manuscript* file)

**Response**: Dr Pramod Avti, associate Professor, Department of Biophysics, Post Graduate Institute of Medical Education and Research has help us in editing of the manuscript. We are thankful and acknowledged the same in the manuscript.

4. We note that you have indicated that data from this study are available upon request. PLOS only allows data to be available upon request if there are legal or ethical restrictions on sharing data publicly. For information on unacceptable data access restrictions, please see <http://journals.plos.org/plosone/s/data-availability#loc-unacceptable-data-access-restrictions>.

**Response**: We have already published an article on genetic analysis which is accessible in the pubmed portal without any restriction (doi:10.1016/j.ygeno.2020.09.044).

In your revised cover letter, please address the following prompts:

a) If there are ethical or legal restrictions on sharing a de-identified data set, please explain them in detail (e.g., data contain potentially identifying or sensitive patient information) and who has imposed them (e.g., an ethics committee). Please also provide contact information for a data access committee, ethics committee, or other institutional body to which data requests may be sent.

**Response**: Not applicable.

b) If there are no restrictions, please upload the minimal anonymized data set necessary to replicate your study findings as either Supporting Information files or to a stable, public repository and provide us with the relevant URLs, DOIs, or accession numbers. Please see <http://www.bmj.com/content/340/bmj.c181.long> for guidelines on how to de-identify and prepare clinical data for publication. For a list of acceptable repositories, please see <http://journals.plos.org/plosone/s/data-availability#loc-recommended-repositories>.

We will update your Data Availability statement on your behalf to reflect the information you provide.

**Response**: We have recently published an article and could be used as data source for current manuscript (doi: 10.1016/j.ygeno.2020.09.044). Meanwhile, we will initiate the process to upload the data in public repository.

5. Thank you for stating the following in the Acknowledgments Section of your manuscript:

[Department of Biotechnology (No. BT/PR17550/MED/30/1755/2016), New Delhi,
India. The funders had no role in study design, data collection and analysis, decision to publish, or preparation of the manuscript.]

We note that you have provided funding information that is not currently declared in your Funding Statement. However, funding information should not appear in the Acknowledgments section or other areas of your manuscript. We will only publish funding information present in the Funding Statement section of the online submission form.

Please remove any funding-related text from the manuscript and let us know how you would like to update your Funding Statement. Currently, your Funding Statement reads as follows:

 [The funders had no role in study design, data collection and analysis, decision to publish, or preparation of the manuscript.]

**Response**: We have removed the funding statement from the manuscript as per the suggestion of the reviewer.

Please include your amended statements within your cover letter; we will change the online submission form on your behalf.

**Response**: Please write the funding statement as “Department of Biotechnology (No. BT/PR17550/MED/30/1755/2016), New Delhi, India and no role in study design, data collection and analysis, decision to publish, or preparation of the manuscript”.

Reviewers' comments:

Reviewer's Responses to Questions

Reviewer #1: The manuscript is interesting and well organized. The methods selected and applied for the analyzes are correct and adequate. As the rationale of the paper evaluates "Sleeping pattern and activities of daily living" it is also crucial to discuss the possible role of epigenetics in the onset and progression of AMD. Numerous papers have been published in the literature describing a possible role of epigenetic mechanisms in the etiopathogenesis of the disease. This topic should be included in both the introduction and discussion with the aim of improving the originality of the manuscript.
To date, when studying multifactorial diseases it is very important to take into account the role of epigenetics.

**Response**: Thanks for valuable comment. We have updated the manuscript by discussing the significance of epigenetic modification and its implication in genetic and pathological complexity of AMD. Please see introduction and discussion sections of the manuscript.
